# Snapshot Study of Whole Genome Sequences of *Escherichia coli* from Healthy Companion Animals, Livestock, Wildlife, Humans and Food in Italy

**DOI:** 10.3390/antibiotics9110782

**Published:** 2020-11-06

**Authors:** Elisa Massella, Cameron J. Reid, Max L. Cummins, Kay Anantanawat, Tiziana Zingali, Andrea Serraino, Silvia Piva, Federica Giacometti, Steven P. Djordjevic

**Affiliations:** 1 Department of Veterinary Medical Sciences, University of Bologna, 40126 Bologna, Italy; elisa.massella@libero.it (E.M.); andrea.serraino@unibo.it (A.S.); silvia.piva@unibo.it (S.P.); federica.giacometti3@unibo.it (F.G.); 2 The ithree Institute, University of Technology Sydney, City Campus, Ultimo, NSW 2007, Australia; cameron.reid@uts.edu.au (C.J.R.); max.cummins@uts.edu.au (M.L.C.); kay.anantanawat@uts.edu.au (K.A.); tizianazingali@gmail.com (T.Z.)

**Keywords:** commensal *Escherichia coli*, antimicrobial resistance, whole genome sequencing, genomic epidemiology

## Abstract

Animals, humans and food are all interconnected sources of antimicrobial resistance (AMR), allowing extensive and rapid exchange of AMR bacteria and genes. Whole genome sequencing (WGS) was used to characterize 279 *Escherichia coli* isolates obtained from animals (livestock, companion animals, wildlife), food and humans in Italy. *E. coli* predominantly belonged to commensal phylogroups B1 (46.6%) and A (29%) using the original Clermont criteria. One hundred and thirty-six sequence types (STs) were observed, including different pandemic (ST69, ST95, ST131) and emerging (ST10, ST23, ST58, ST117, ST405, ST648) extraintestinal pathogenic *Escherichia coli* (ExPEC) lineages. Eight antimicrobial resistance genes (ARGs) and five chromosomal mutations conferring resistance to highest priority critically important antimicrobials (HP-CIAs) were identified (*qnrS1*, *qnrB19*, *mcr-1*, *bla*_CTX-M1,15,55_, *bla*_CMY-2_, *gyrA/parC/parE, ampC* and *pmrB*). Twenty-two class 1 integron arrangements in 34 strains were characterized and 11 ARGs were designated as *intI1* related gene cassettes (*aadA1*, *aadA2*, *aadA5*, *aad23*, *ant2_Ia*, *dfrA1*, *dfrA7*, *dfrA14*, *dfrA12*, *dfrA17*, *cmlA1*). Notably, most *intI1* positive strains belonged to rabbit (38%) and poultry (24%) sources. Three rabbit samples carried the *mcr-1* colistin resistance gene in association with IS*6* family insertion elements. Poultry meat harbored some of the most prominent ExPEC STs, including ST131, ST69, ST10, ST23, and ST117. Wildlife showed a high average number of virulence-associated genes (VAGs) (mean = 10), mostly associated with an ExPEC pathotype and some predominant ExPEC lineages (ST23, ST117, ST648) were identified.

## 1. Introduction

Antimicrobial resistance has been recognized as one of the world’s most pressing public health problems, with implications for human and veterinary medicine, wildlife and environmental ecosystems. In recent decades we have witnessed a dramatic spread and diffusion of multidrug resistant (MDR) pathogens. In the context of human medicine, the decrease in bacterial susceptibility to critically important antimicrobials (CIAs) and the quick diffusion of extended-spectrum beta lactamase (ESBL) producers are a major concern [1].

The consequences of MDR are particularly severe and characterized by an increase in infection severity, treatment failure, hospitalizations and mortality, with growing costs for health care. It has been estimated that AMR causes 700,000 deaths every year globally, with 25,000 victims and over €1.5 billion in healthcare costs in the European Union (EU) [2].

Horizontal gene transfer (HGT) plays a primary role in the exchange of genetic material amongst bacteria and allows them to evolve and adapt rapidly to novel selective pressures. HGT of ARGs is mediated by mobile genetic elements (MGEs) and facilitated by the interconnection between bacteria, their hosts and the environment [3]. Animals (livestock, companion animals, wildlife), the community, hospital and industrial settings (including food-production) have been investigated as possible sources of AMR [4,5,6]. The interaction between these environments is complex and understanding vectors of AMR transfer is a central tenet in mitigation efforts to limit its impact. Municipal, agricultural and industrial wastewater, aquaculture, animal manure and sewage sludge have been recognized as major pathways of AMR transmission between different ecosystems [7,8]. Data describing spatial and temporal trends of AMR carriage are essential for epidemiological evaluation and preventive measure implementation. In this context, the European Union has defined AMR as a special health issue to be monitored by epidemiological surveillance systems [9].

*Escherichia coli* (*E. coli*) are commensal inhabitants of the gastrointestinal tract of mammals and most vertebrate species, can proliferate in diverse aquatic environments and are often resistant to multiple antibiotics. *E. coli*: (i) exists in feces as a commensal or as a pathogen and is frequently exposed to antimicrobial selection pressures; (ii) can cause intestinal and extraintestinal disease in humans and animals; and (iii) lives freely in water and colonizes wild animal vectors such as birds and insects [10,11]. Because of these features, combined with the capacity to acquire genetic material via HGT, *E. coli* is a bacterium of choice for studying AMR from a One Health perspective [12]. 

From a global perspective, extensive genomic evaluation of ARGs, VAGs and their association with MGEs in nonclinical *E. coli* is generally lacking. Most studies have typically relied on phenotypic AMR and molecular analyses of pathogens [13,14,15,16]. Prior to WGS, genotypic evaluation was mainly performed by polymerase chain reaction (PCR) without considering the often mosaic and complex mobile antimicrobial genetic structures involved. This is particularly evident in Italian studies [17,18,19,20]. However, in recent years WGS has been increasingly used to examine drug-resistant commensal and pathogenic *E. coli* from humans [21,22,23], food animals [24,25,26], fresh produce [26,27], companion animals [28], wildlife [29], wastewater [30] and in pristine aquatic environments [31]. Despite these efforts, an exhaustive knowledge of AMR mechanisms, sources and epidemiology is still lacking.

Italy in particular, currently lacks this data, rarely performing WGS to characterise AMR in *E. coli* indicator from human and animal sources or from food and wildlife.

Here, we present WGS of 279 commensal *E. coli,* isolated from different sources (including livestock, companion animals, wildlife, food and humans) in Italy. We provide data related to their phylogenetic diversity, carriage of ARGs, VAGs, biocide resistance genes and their association with MGEs.

## 2. Results

The original collection consisted of 288 *E. coli* strains. Nine of these (EM1_Be20, EM1_Ca14, EM1_Fs21, EM1_Rb15, EM1_Rb25, EM1_Ve16, WM1_Wb2, EM1_Wb24, EM1_Wb25) were excluded due to PCR misidentification or inadequate DNA quality. Therefore, the final study collection consisted of 279 *E. coli* (25 poultry; 25 swine; 25 dairy; 25 human; 25 mollusc; 25 wild animal; 24 beef; 24 vegetable; 24 fish; 23 rabbit; 22 wild boar; 12 companion animal).

### 2.1. In Silico Typing and Phylogeny

A total of 136 sequence types were identified, with ST10 (16; 5.7%), ST155, ST847 (both 9; 3.2%), ST69 (8; 2.9%), ST20 (7; 2.5%), ST23, ST117, ST216 (all 6; 2.2%) being the most common. Ninety-five sequence types were represented by a single isolate. Most sources displayed a diversity of STs, though some exhibited clonality including beef, swine and poultry with eight ST847, six ST10 and five ST117, respectively.

One hundred and fifty-eight different serotypes were predicted for the 279 strains analyzed. Forty-four strains were O nontypable with 20 different H types, meanwhile for one O107 isolate, the H type was not determined. Serotype variability was pronounced among sources, except for human sources with six O1:H7 strains (4, ST59; 2, ST95).

The most common phylogroup identified among the collection was B1 (130; 46.6%), followed by A (81, 29%) D (47; 16.8%) and B2 (21; 7.5%). Most beef, companion animal, mollusc, rabbit, vegetable and wild animal strains (≥50%) belonged to phylogroup B1. Meanwhile phylogroup A was the most represented (≥40%) in dairy, fishery, poultry and swine sources. Phylogroup D was prevalent in human (12/25; 48%) and wild boar (11/22; 50%) isolates. Interestingly, wild boar and wild animal sources contained the highest number of B2 strains (6 and 3, respectively), which are usually associated with pathogenicity.

**Figure 1:**
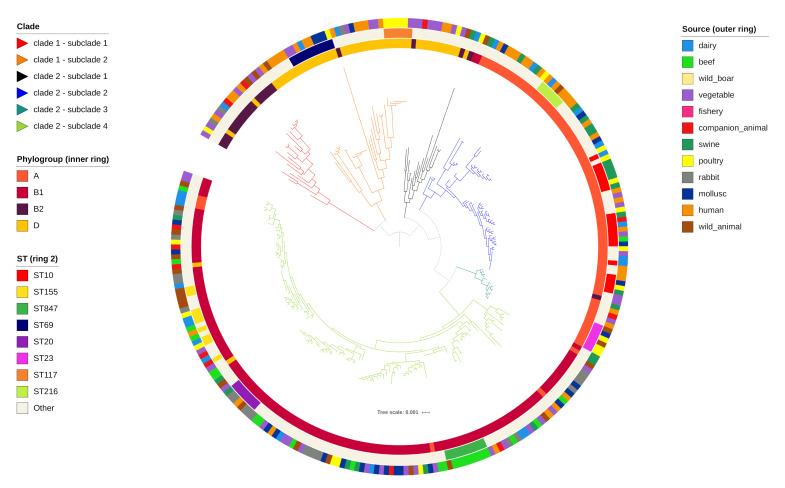
A mid-point rooted, maximum-likelihood phylogenetic tree of 279 commensal *E. coli* included in the study. Branches are colored by clade and subclade according to the legend. Phylogroups (inner ring), sequence types (middle ring) and sources (outer ring) are annotated according to the legend.

Phylosift analysis produced a maximum-likelihood tree (Figure 1) with the first major split separating phylogroups B2 and D from A and B1. Tree topology was highly congruent with Achtman MLST and generally congruent with phylogroup distribution. Clade 1 was composed of two subclades, one mostly comprising phylogroup B2 and one primarily phylogroup D. ST69 was the prevalent sequence type of clade 1 with eight strains. Clade 2 was split into four subclades, the first mostly containing phylogroup D, the second and third were primarily phylogroup A and the fourth was dominated by phylogroup B1. The most common lineage of clade 2 was ST10 (16) all belonging to subclade 2. Strains of the same source clustered on distinct branches in some cases. However, sources were generally distributed across multiple clades.

### 2.2. Antimicrobial Resistance Genes

**Figure 2:**
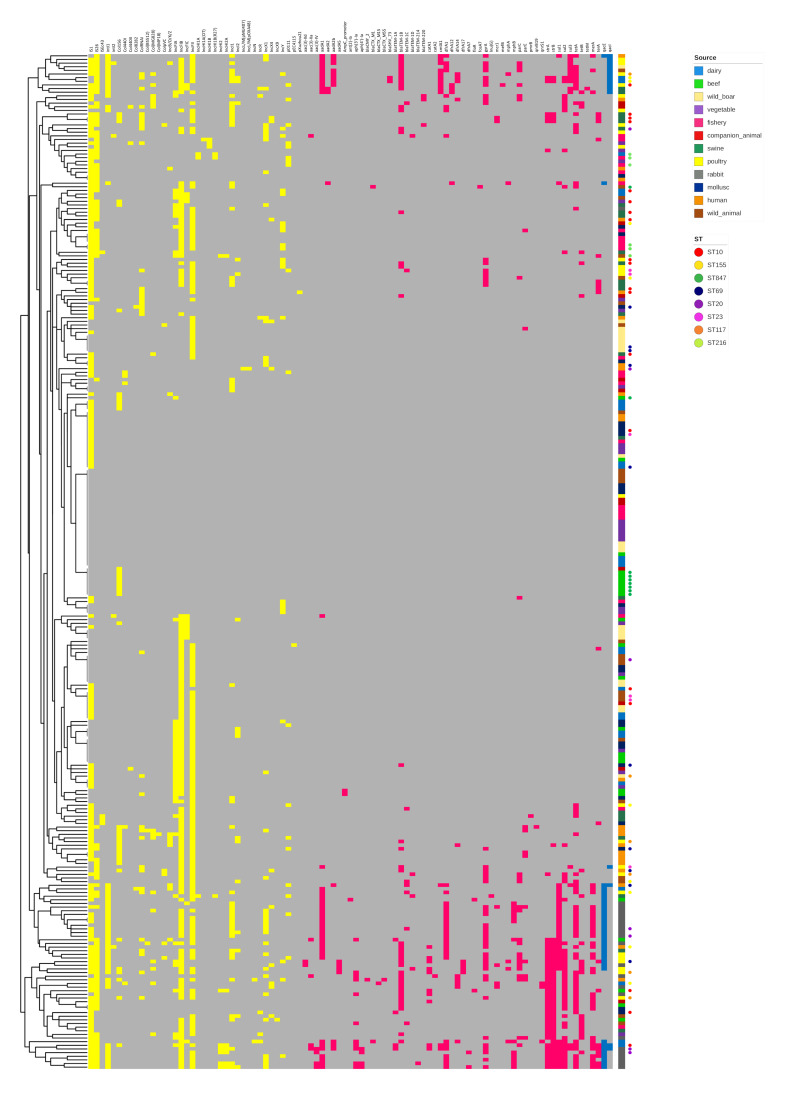
Heat map depicting carriage of mobile genetic elements (MGEs, yellow), antimicrobial resistance genes (ARGs red) and plasmid mediated quarternary ammonium compound (QAC) resistance genes (blue) in the strain collection. Grey indicates absence. The dendrogram on the left represents clustering of *E. coli* isolates according to genetic profile similarities.

**Figure 3:**
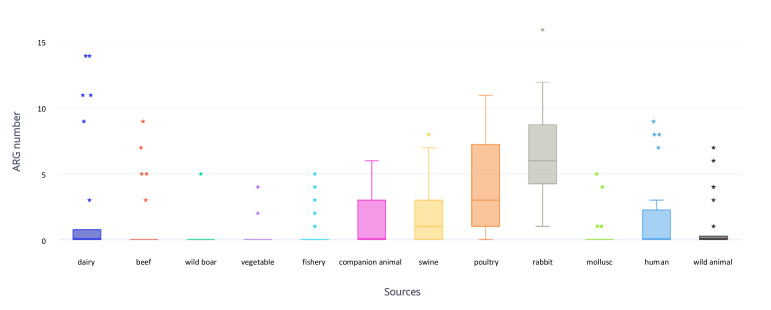
Box plot representing ARG number and distribution in the different sources considered in the study (poultry, swine, human, mollusc, wild animal, dairy, n = 25; beef, vegetable and fishery, n = 24; rabbit, n = 23; wild boar, n = 22; companion animal, n = 12). ⋆: outlier.

One hundred and seventy-nine strains (64.2%) did not carry ARGs. The remaining 100 (35.8%) isolates carried between one and 16 ARGs, with 77 (71.3%) strains containing at least three. Among the latter, 43/77 (55.8%) strains belonged to phylogroup B1, 20/77 (26%) to phylogroup A, 12/77 (15.6%) to phylogroup D and 2/77 (26%) to phylogroup B2. Interestingly, most strains carrying ≥10 ARGs belonged to phylogroups B1 (6/11) and A (3/11). Forty-six different ARGs were identified in our collection (Figure 2), with tetracycline resistance gene *tetA* (57/279; 20.4%), sulfonamide resistance gene *sul2* (45/279; 16.1%), penicillin resistance gene *bla_TEM-1b_* (43/279; 15.4%) and streptomycin resistance genes *strA/B* (42/279; 15.1%) being the most common. Colistin resistance gene *mcr-1* was discovered in six (2.2%) strains, namely three rabbit, two swine and one dairy strain. Genes encoding extended spectrum beta-lactamases (*bla_CTX-M-1_, bla_CTX-M-15_, bla_CTX-M-55_*) were identified in 4 (1.4%) isolates, namely two dairy, one wild animal and one human. All ESBL strains carried a high number of ARGs (mean = 9) and VAGs (mean = 16). AmpC beta lactamase gene *bla_CMY-2_* was identified in one human isolate. Fluoroquinolone resistance genes (*qnrS1* and *qnrB19*) were discovered in two dairy, one human and one rabbit strain. The mean number of ARGs present in each source was: rabbit eight; poultry four; dairy three; swine and human two each; wild animal, beef, companion animal, mollusc and fishery, one each. The sources carrying the lowest number of ARGs were vegetable and wild boar, with only two and one resistant strains, respectively (Figure 3). Mutations in *gyrA, parC* and *parE* genes, conferring presumptive fluoroquinolone resistance, were identified in 61 strains, mostly of rabbit (17/54), poultry (13/54) and human (9/54) origin. Point mutation in the AmpC promoter, associated with hypothetical AmpC-type cephalosporinase expression, was identified in two beef strains. A single swine isolate contained SNPs in *pmrB*, conferring presumptive resistance to colistin. Heavy metal resistance genes *merA* and *terA,* conferring resistance to mercury and tellurium were identified in 34 and 19 isolates, respectively.

### 2.3. Virulence-Associated Genes

**Figure 4:**
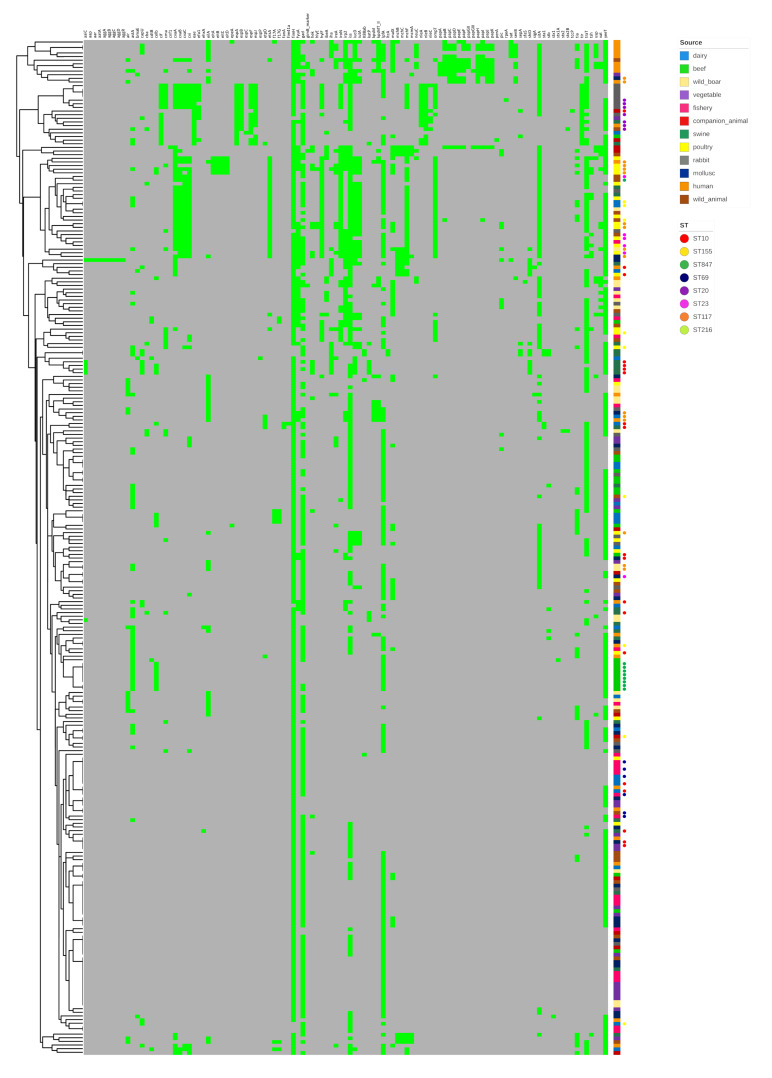
Heat map depicting VAG carriage (light green) in the strain collection. Grey indicates absence. The dendrogram on the left represents clustering of *E. coli* isolates according to genetic profile similarities.

**Figure 5:**
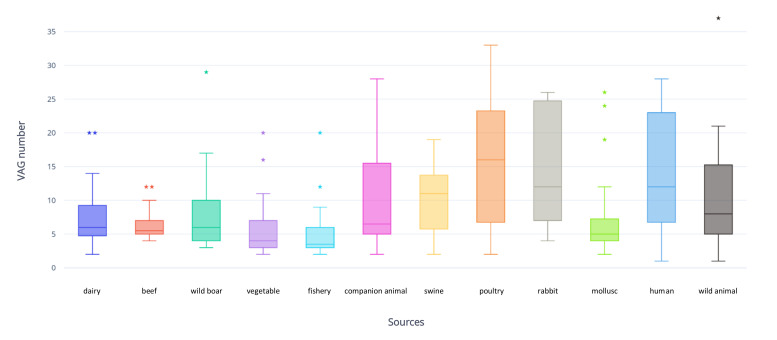
Box plot representing VAG number and distribution in the different sources considered in the study (poultry, swine, human, mollusc, wild animal, dairy, n = 25; beef, vegetable and fishery, n = 24; rabbit, n = 23; wild boar, n = 22; companion animal, n = 12). ⋆: outlier.

The whole collection was screened for VAGs associated with ExPEC and intestinal pathogenic *E. coli* (IPEC) pathotypes. One hundred and eleven different VAGs were identified (Figure 4). All strains carried between one and 37 VAGs. A high number (≥10) of virulence determinants were identified in 102 (36.6%) isolates, most of them belonging to phylogroup B1 (41, 40.2%), followed by D (27; 26.5%), A (21, 20.6%) and B2 (13; 12.7%). Most VAGs were typical of ExPEC pathotype, including different genes encoding for adhesins (*fimH, pap, iha, bmaE, sfaS*), invasins (*ibeA*), iron acquisition systems (*iucD, iutA, fyuA, irp2, iroN, ireA, tsh, sitA*), toxins (*hlyE, cnf1, cdtB, usp, sat, picU, vat*) and protectins (*kpsMT-II, traT, ompT, iss, cvaC*). Both ExPEC and IPEC VAGs were identified in four ST20 and four ST40 strains from rabbits. The mean number of VAGs for each source was: poultry and human, 15 each; rabbit, 14; companion animal and wild animal, 11 each; swine, 10; wild boar, dairy, mollusc, 8 each; beef and vegetable, 6 each; fishery, 5 (Figure 5). Three strains (1.1%) carried ≥30 VAGs, including two from poultry (1, ST117; 1, ST131) and one ST648 from a wild animal (stork). 

### 2.4. Class 1 Integron Structures

Short read screening identified the *intI1* class 1 integrase gene in 50/279 (17.9%) strains, mostly present in phylogroup B1 (31/50; 62%), followed by A and D (9/50; 18% each) and B2 (1/50; 2%).

**Figure 6:**
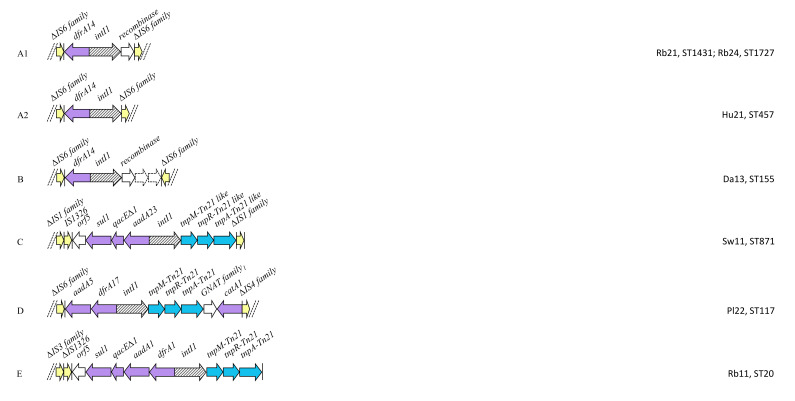

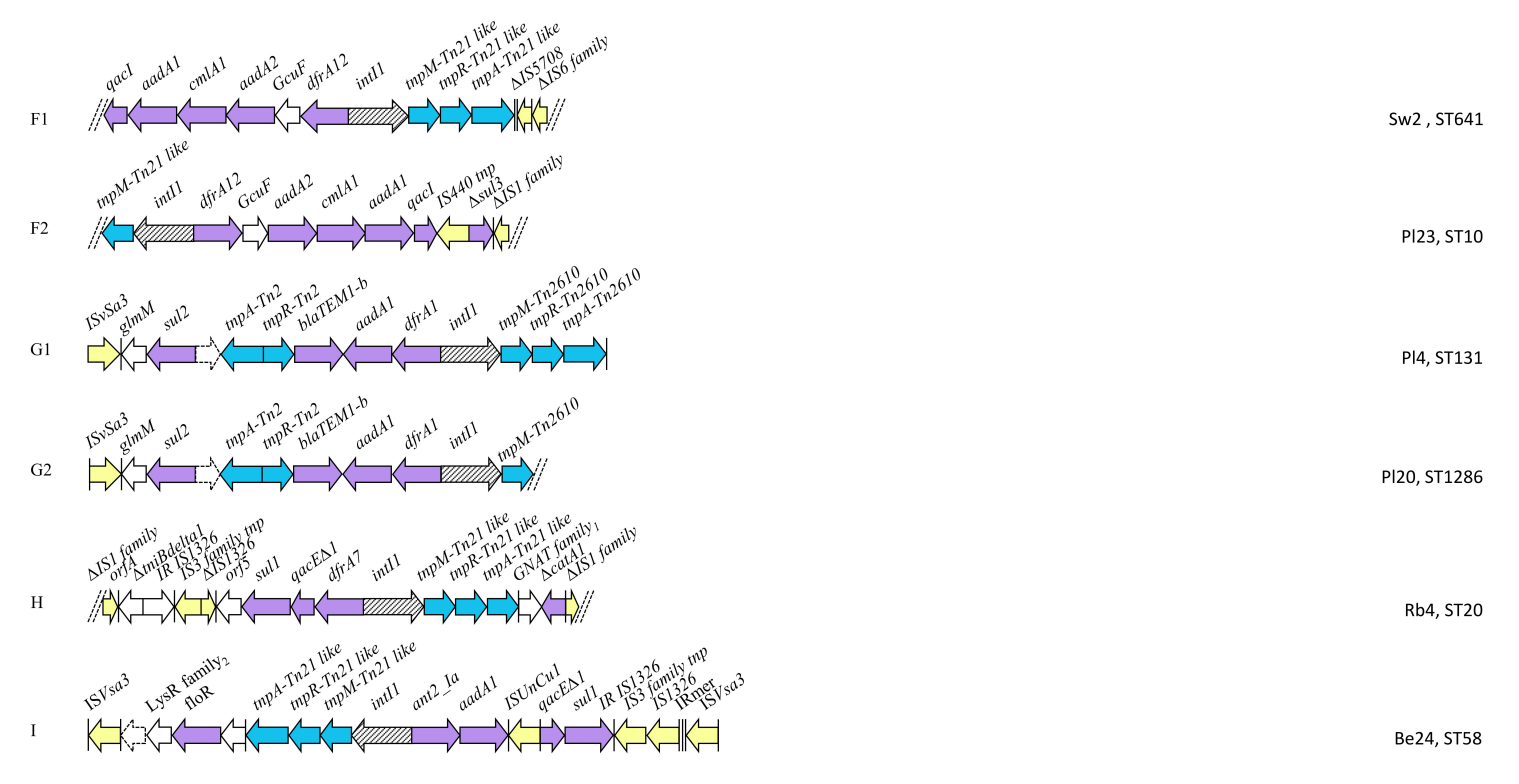

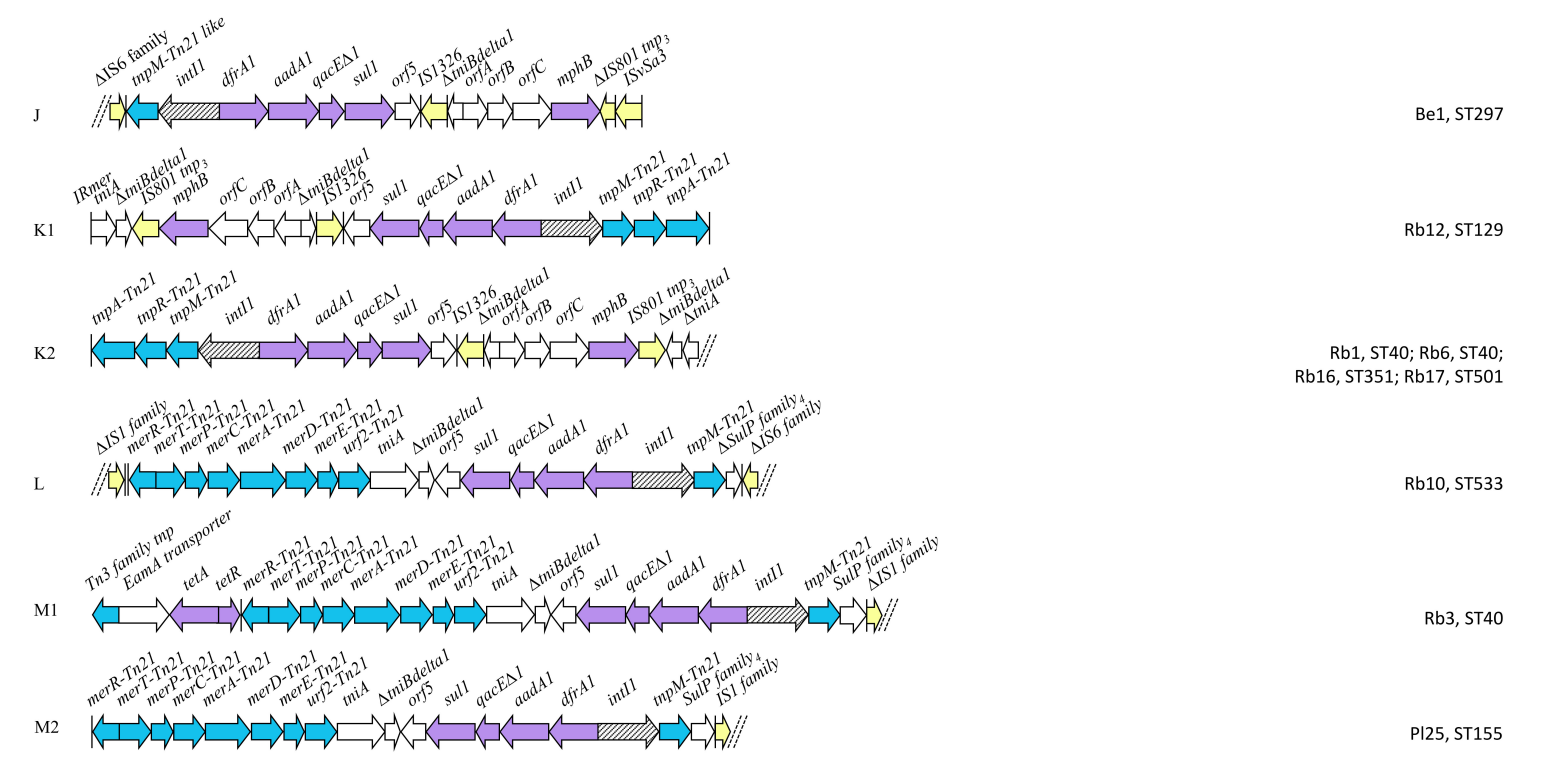

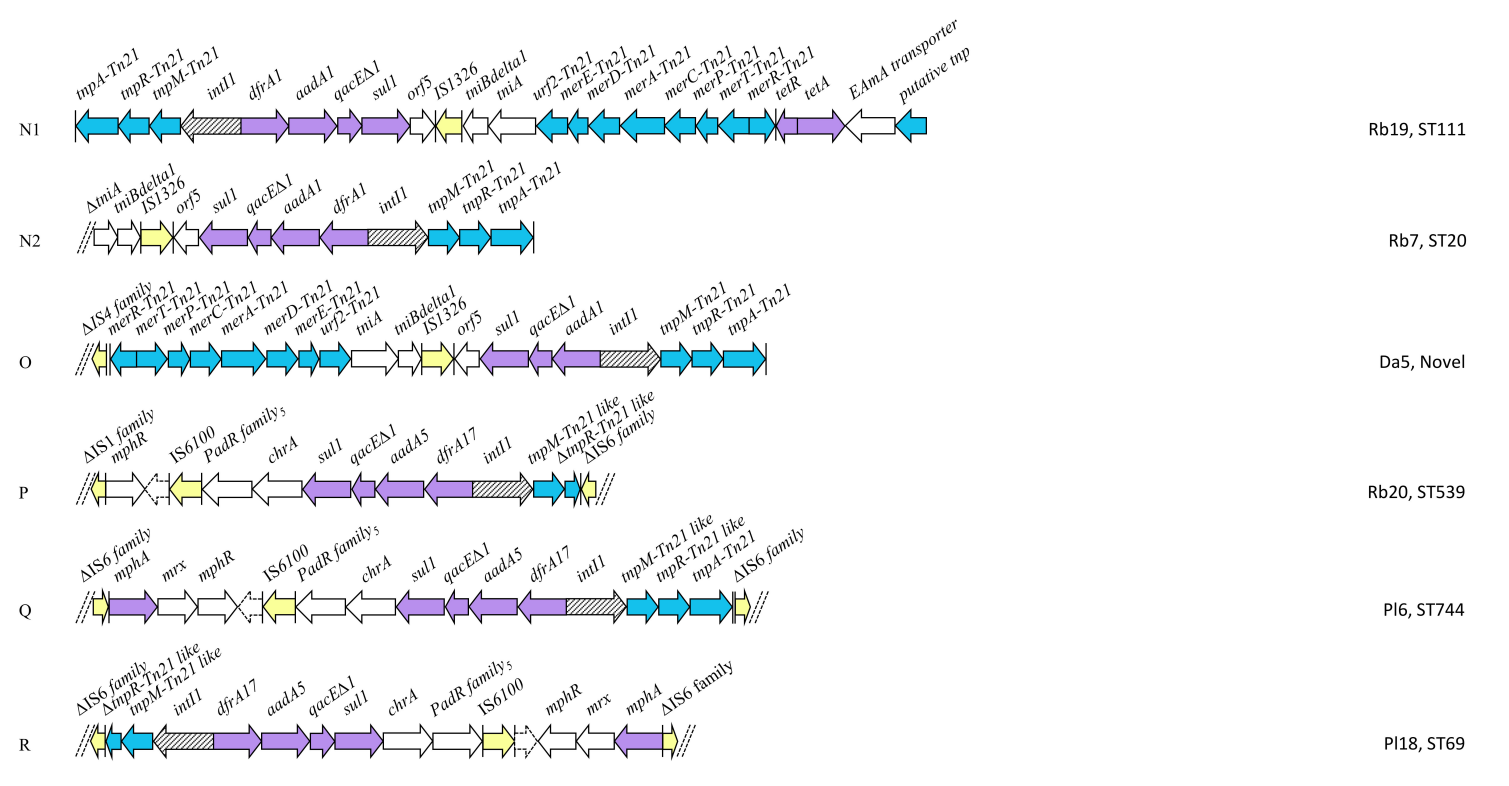

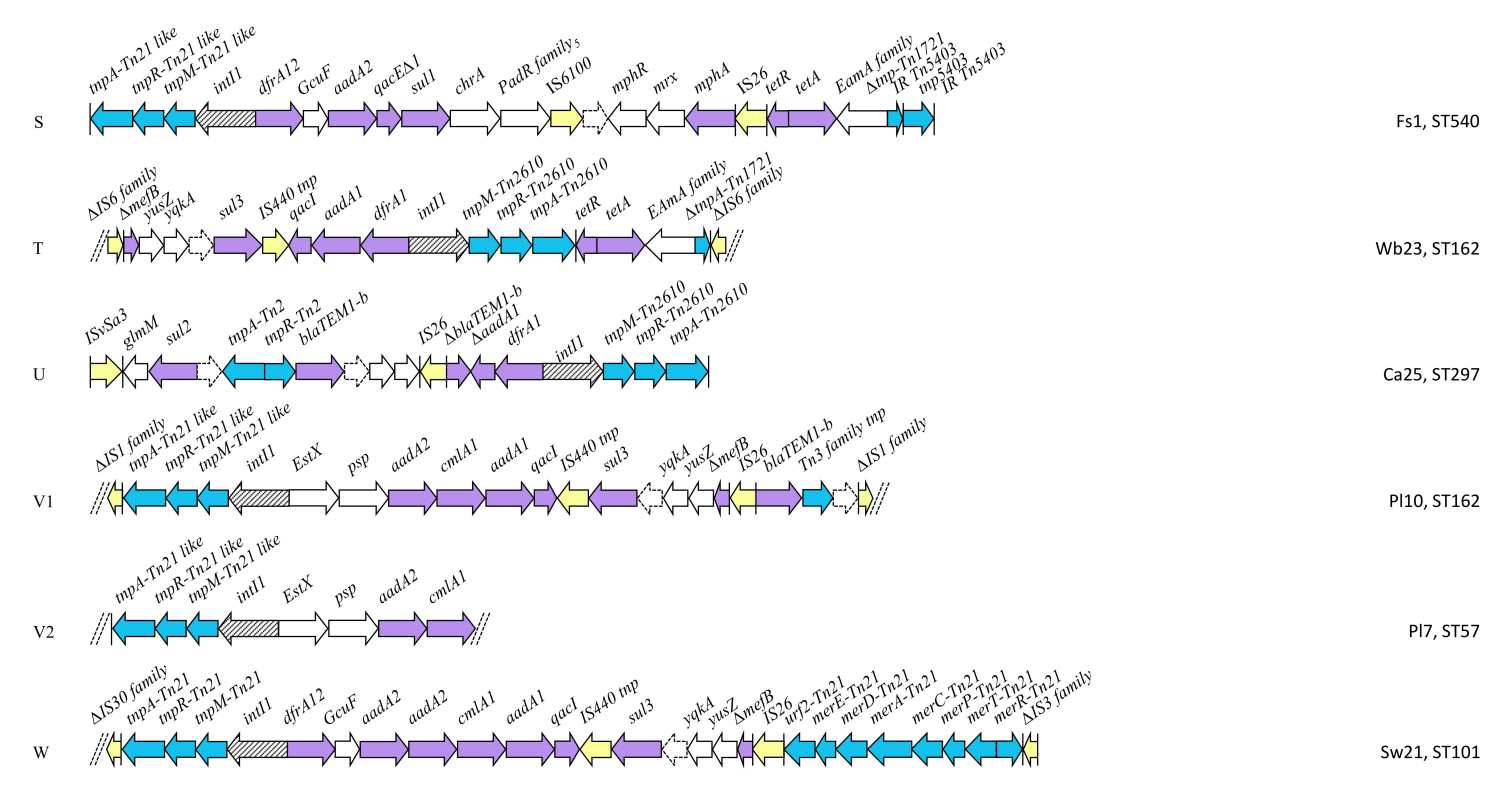
Schematic diagram (not to scale) of integron structures identified in 34 commensal *E. coli*. Arrows represent ORFs. Arrows with broken lines indicate hypothetical proteins. Vertical bars represent inverted repeats. Dashed double diagonal lines represent sequence scaffold breaks. Elements displayed are color coded (ARGs = purple; IS elements = yellow; transposon elements = light blue). 1: N-acetyltransferase, GNAT family; 2: transcriptional regulator, LysR family; 3: homologue of IS801 transposase like protein of *Pseudomonas pseudoalcaligenes*; 4: SulP family inorganic anion transporter; 5: PadR family transcriptional regulator; orfA: homologue of penicillin-binding protein 4 of *Streptomyces lactamdurans*; orfB: homologue of AcrR potential operon repressor; orfC: homologue of RdmC protein of *Streptomyces purpurascens.*

Following de novo assembly, we identified 44 strains with scaffolds carrying a complete *intI1* gene. Thiry-four of 44 strains carried cassette array genes and were annotated in order to characterize the different integron structures present in the collection. Twenty-three arrangements were characterized and designated letters (A-W). Derivatives of (A-W) were named with the letter of the principal structure followed by a number (Figure 6). Eleven ARGs were identified as *intI1* related gene cassettes, namely those conferring resistance to aminoglycosides; *aadA1, aadA2, aadA5, aad23, ant2_Ia*, trimethoprim; *dfrA1, dfrA7, dfrA12, dfrA14, dfrA17*, and chloramphenicol; *cmlA1*. The most common integron cassette array was *aadA1-dfrA1*, present in 14/35 (40%) strains. Both *sul1* and *sul3* were identified in the 3`-CS of characterized integrons. Two isolates (one human and one poultry strains) harbored both *intI1* and *intI2* integrase genes.

### 2.5. Antibiotic Resistance Gene Carriage in Strains Carrying intI1 Compared with Those That Do Not Carry intI1

**Figure 7:**
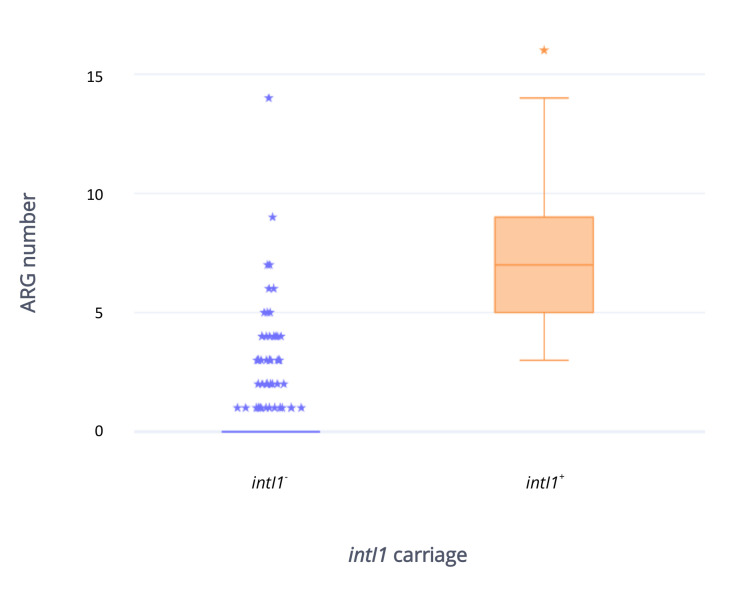
Box plot representing ARGs number and distribution in *intI1*^+^ (n = 50) and *intI1*^−^ (n = 229) strains considered in the study. ⋆: outlier.

**Figure 8:**
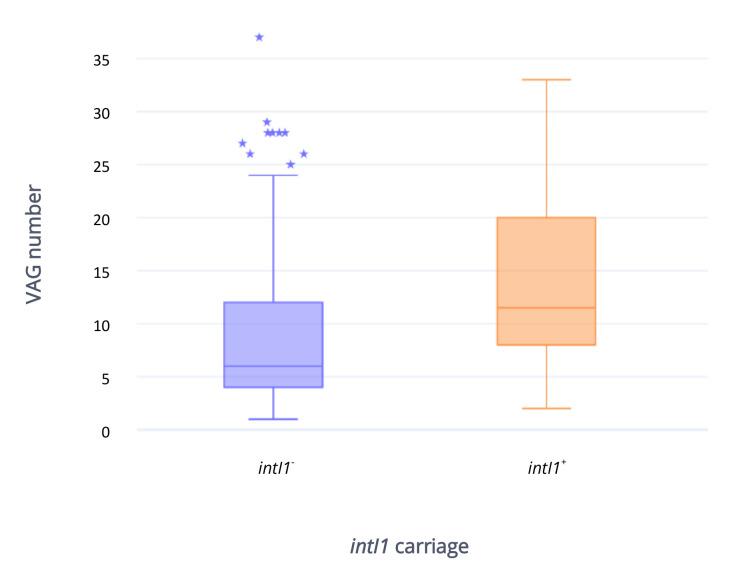
Box plot representing VAGs number and distribution in *intI1*^+^ (n = 50) and *intI1*^−^ (n = 229) strains considered in the study. ⋆: outlier.

Short read sequencing indicated that 50/279 (17.9%) strains carried *intI1* (*intI1*^+^), most of them isolated from food-producing animals (19/50; 38%) and related food (24/50; 48%). In particular, rabbit (19/50; 38%) and poultry (12/50; 24%) sources were the most represented, followed by four human (8%), two wild animal (4%), one companion animal (2%) and one fishery (2%). As expected, a higher number of ARGs was harbored by *intI1*^+^ strains compared to strains that were *intI1*^−^, with a mean of seven to one respectively (Figure 7). ARGs which are usually not a part of the typical class 1 integron structure, including *tetA* (*intI1*^+^, 42/50, 84%; *intI1*^−^ 34/229, 14.8%), bla_TEM_ (*intI1*^+^ 33/50, 66%; *intI1*^−^ 30/229, 13.1%) and *strA/B* (*intI1*^+^, 22/50, 44%; *intI1*^−^ 22/229, 9.6%), were present in both groups. As expected, aminoglycoside (*aadA*) and trimethoprim (*dfr*) resistant determinants, widely observed as gene cassettes, were widespread among *intI1*^+^ strains (43/50, 86% and 46/50, 92% respectively) and rare in *intI1*^−^ isolates (3/229, 1.3% and 5/229, 2.2%, respectively). ESBL, polymyxin, and quinolone resistance genes were rarely identified among *intI1*^+^ (2/50, 4%; 4/50, 8%; 2/50, 4% respectively) and *intI1*^−^ (2/229, 0.9%; 2/229, 0.9%; 2/229, 0.9% respectively) isolates. *bla_CMY-2_* gene was harbored by a single *intI1*^+^ isolate. VAGs appeared to be more common in *intI1*^+^ strains when compared to the *intI1*^−^ group, with means of 14 and 9 respectively (Figure 8). The IncF plasmid replicon was the most frequent in both *intI1*^+^ (47/50, 94%) and *intI1*^−^ (135/229, 59%) strains. Notably, pandemic (ST69, ST95, ST131) and emerging (ST10, ST23 ST58, ST117, ST405, ST648) ExPEC lineages were present in both groups (*intI1*^+^ 8/50, 16%; *intI1*, 37/229, 16.2%).

### 2.6. Plasmid Incompatibility Groups

Thirty-three different plasmid replicons (Figure 2) were identified in the collection. IncF was the most common (182/279; 65.2%), whilst IncI (57/279; 20.4%) and IncX (45/279; 16.1%) were also frequently observed. These replicons were present across multiple sequence types. 

### 2.7. Biocide Resistance Genes

Our collection was screened for different efflux pump genes and related regulators linked with the extrusion of a wide variety of molecules, including biocides and antimicrobials. Most genetic determinants, known to be chromosomally encoded, were widespread. Genes involved in quaternary ammonium compound resistance (*emrE, mdfA*), oxidative stress tolerance and protection/peroxygens resistance (*ibpA, ibpB, sodA, sodB, ydeI, ymgB*) and phenolic compound resistance (*acrAB, fabI*) were frequently identified. Integron-associated *qacE* and *qacI* genes were mostly identified in rabbit (17) and poultry (10) sources, alone or in combination (Figure 2).

Complete VAG, ARG, MGE and efflux pump/biocide resistant gene carriage data are available in Supplementary Materials (Tables S1, S3 and S4).

## 3. Discussion

Using WGS we characterized 279 *E. coli* recovered from diverse animal and food sources in Italy to garner insight into the diversity of *E. coli* sequence types and the antimicrobial resistance and virulence genes they carry. We observed 136 *E. coli* STs, including pandemic (ST69, ST95, ST131) and emerging (ST10, ST23, ST58, ST117, ST405, ST648) EXPEC lineages. Most of the strains did not carry class 1 integrons but *intI1*^+^ isolates carried more ARGs compared to those that were *intI1*^−^. Class 1 integron structure analysis among 50/279 (17.9%) *intI1*^+^ strains identified 22 class 1 integron variants among 34 structures. Limitations of short read sequence data precluded analysis of the remaining 16 class 1 integrons. ARGs that conferred resistance to HP-CIAs included *qnrS1*, *qnrB19*, *mcr-1*, *bla*_CTX-M1,15,55_, and *bla*_CMY-2_. Chromosomal mutations in *gyrA/parC/parE* were notable in poultry and rabbit sources.

### 3.1. Antimicrobial Resistance

The most common ARGs in all sources were *tet* (mostly *tetA*, 57/279), *sul* (mostly *sul2*, 45/279) and *bla*_TEM_ (mostly *bla*_TEM-1b_, 43/279). These genes are generally reflective of common phenotypic resistance profiles of commensal *E. coli* of animal, food and human origin previously reported in Europe [12,18,32,33,34,35,36]. The *tet*–*sul*–*bla*_TEM_ genetic profile in livestock (cattle, swine, poultry, rabbit) reflects resistance to the most commonly used antimicrobials in Italian livestock production [37]. This profile was also observed in environments not commonly associated with direct antimicrobial selective pressure, like aquaculture (molluscs and fish), vegetables and wildlife. Resistance to tetracycline, sulfonamides and beta-lactams has been reported in aquaculture [35,38] and vegetables [36,39,40] in Europe. However, Italian data about AMR are lacking, probably due to the rare or absent antimicrobial use in these sectors [41]. Hence, a proper comparison between our findings and previous phenotypic reports in these sources is difficult to perform. A similar phenotypic AMR profile has been previously reported in wildlife [42,43], considered an AMR bioindicator [42,44,45] primarily because related AMR is strictly influenced by livestock and human density/activity [46,47].

The consistency of AMR profile identified in aquaculture, vegetables and wildlife with those observed in livestock, humans and companion animals supports AMR diffusion from settings with high antimicrobial use. Fecal contamination is considered the major path for AMR bacteria, genes and antimicrobial residues diffusion [7,8,32,48,49]. Sewage and manure contaminate groundwater and aquatic systems. Therefore, irrigation water and manure-based fertilizers may act as carriers of AMR bacteria and genes, polluting agricultural production [36,50]. Similarly, sewage and runoff from land could be responsible for AMR observed in aquaculture systems [51,52,53].

Streptomycin resistance genes *strA/B* were frequently observed (42/279; 15%). Our findings are generally consistent with previous studies [18,32,35,36,54,55], barring the low levels of phenotypic aminoglycoside co-resistance typically reported in swine, poultry and beef [12,33,56,57]. Currently, streptomycin has limited therapeutic usage in both humans and animals in Europe [37,58]. However, *strA/B* are common streptomycin determinants among Enterobacteriaceae isolated from humans and animals [25,59]. *strA/B* are usually genetically linked and have been frequently associated with Tn*5393* [60] and Tn*6029*/Tn*6026* [61] on different multi-resistance plasmids (including IncH1, IncH2, IncHII, IncZ, IncN, IncQ, IncU) [62], circulating in bacterial populations. Moreover, these genes are often clustered on different plasmids with the *sul2* gene [63,64], encoding resistance to some of the most frequently sold antimicrobials for livestock. The abundance of *strA/B* might, therefore, be explained by co-selection.

Cephalosporins (third, fourth and fifth generation), polymyxins and quinolones are considered HP-CIAs, the last-line of treatment for serious human infections. ESBL and polymixin ARGs, and *pmr* chromosomal mutations were detected in low frequency among the collection and were mostly identified in food-producing animals and related food. Our findings are in accordance with low cephalosporin and colistin phenotypic resistance identified in *E. coli* from livestock and related meat in Europe [12,18,33,35,56,57]. Nonetheless, *mcr-1* and *mcr-2* colistin resistance genes have been reported in European *E. coli* from poultry in Romania [65] and poultry and swine in Spain [66].

Notably, most ESBL genes (2 *bla*_CTX-M-1_ genes in wild animal and dairy strains; 1 *bla*_CTX-M-15_ in a dairy strain; 1 *bla*_CTX-M-55_ in a human strain) were found in proximity to IS*Ecp1*. IS*Ecp1* is a member of the IS*1380* family and has previously been associated with ESBL genes [67,68]. A single copy of this IS element is able to mobilize downstream genes through transposition [69,70]. De novo assemblies revealed that *mcr-1* was flanked by IS*6* family members in five out of seven carriers. Although assembly scaffold breaks preclude definitive identification of these IS elements, read-mapping identified IS*26*, a member of the IS*6* family, in these strains (three rabbits and two swine). IS*26* plays an important role in the evolution and mobilization of ARGs worldwide. These findings suggest a possible involvement of IS*26* in the spread of *mcr-1*. Further studies are necessary to establish the precise IS elements involved and their effective ability to transmit *mcr-1*.

Fluoroquinolones and quinolones are important antimicrobials used in both hospital and community settings, with only a minor role in companion and food-producing animals [37,58]. Contrastingly, they are reported as some of the most used antimicrobials in poultry and rabbit production systems in Europe [41] despite the European Medicines Agency (EMA) not supplying stratified sales data of veterinary antimicrobials by food-producing animal species [37]. In recent years, there has been a significant increase in fluoroquinolone resistance in human clinical *E. coli* in Europe, with the highest resistance rates observed in Italy [71]. On the contrary, low resistance to these antimicrobials has been usually observed among livestock in the EU, with the exception of poultry [12,33]. In our study, quinolone/fluoroquinolone resistance was mainly identified in poultry (13) and rabbit (15) sources and arose due to chromosomal mutations in *gyrA*/*parC*/*parE* genes. Our findings are in accordance with those previously reported in poultry [33] and rabbits [18] and point to fluoroquinolone usage in these sectors.

Heavy metal resistance genes *merA* and *terA* identified in the collection are known to be genetically linked to a variety of AMR genes on Tn*21-* and *Tn1721-*like transposons (*merA*) and large MDR IncHI2 plasmids (*terA*) [72,73]. This likely explains the observation that strains carrying *merA* carried more than three times as many ARGs on average (7.29 vs. 1.87) compared to the whole collection, whilst five rabbit isolates carrying both *merA* and *terA* had an average of 12.4 ARGs, more than six times the collection average. Environmental heavy metal contamination may, therefore, continue to select for these MDR strains even if antimicrobial use in the sector were to cease.

### 3.2. Virulence-Associated Genes

We identified a surprisingly high number of VAGs (in particular in poultry, rabbit and human niches) despite the fact that our study focused on commensal and environmental *E. coli*. Furthermore, genetic virulence patterns indicated the presence of potential ExPEC pathotypes. This is rather concerning since *E. coli* is the most common causative agent of urinary tract infection (UTI) and bloodstream infection (BSI) globally [74,75]. This assumption was strengthened by the identification of different sequence types, recognized as pandemic (ST69, ST95, ST131) [74] or emergent ExPEC lineages (ST10, ST23 ST58, ST117, ST405, ST648) [76]. 

*qacE* and *qacI* genes, known to be associated with class 1 integron structure, were identified primarily within *intI1^+^* sequences [77,78,79,80]. *qacE* and *qacI* encode small multidrug resistance efflux pump family (SMR) proteins, conferring resistance to quaternary ammonium compounds (QACs), which are commonly used disinfectants in hospitals, healthcare facilities and the food industry [81]. Resistance nodulation division family (RND) efflux pump genes (*acrAB, acrEF, acrD*) were also widely identified in the collection. RND family efflux pumps are involved in clinically significant MDR [82] and may target a wide variety of molecules [82,83,84,85]. The identification of efflux pump genes could suggest potential phenotypic resistance, where antimicrobial determinants are not identified. However, this event is strictly related to efflux pump overexpression genotypes which we did not evaluate [82]. 

### 3.3. intI1^+^ Strains Carry more ARGs and VAGs

Class 1 integrons are a reliable proxy for multiple drug resistance gene carriage [86] and play an essential role in the dissemination and evolution of MDR Gram-negative bacteria. As expected, *intI1*^+^ strains carried more ARGs (mean = 8) than *intI1*^−^ isolates (mean = 1). A wide variety of antimicrobial genetic determinants have been identified as *intI1* related gene cassettes [87]. In particular, nearly half of the characterized integrons (15/34; 44.1%) carried an *aadA1-dfrA1* cassette array. This array is widespread in both commensal and clinical strains isolated from animal and food sources [88]. The most frequent MGEs associated with class 1 integrons in our collection were Tn*21* and close relatives, identified in 26 of 35 (74.3%) *intI1*^+^ strains. Tn*21* transposon and related variants (belonging to the Tn*3* family) are globally disseminated and frequently involved in multiple ARGs and class 1 integron carriage in Enterobacteriaceae [72,89,90]. 

*intI1*^+^ strains carried a higher number of VAGs (mean = 14) compared with *intI1*^−^ strains (mean = 9), and often harbored F plasmid replicons. F incompatibility group members are recognized as the majority of virulence-associated plasmids in *E. coli*. They are known to carry different antimicrobial resistance determinants [91,92] and class 1 integrons [93], creating a concerning combination of virulence and antimicrobial resistance traits. Our findings suggest that ARGs and VAGs may be co-localised on F plasmids in our collection. Long read sequencing data is required to interrogate this hypothesis.

### 3.4. Concerning Sources

Rabbit, poultry and wildlife sources were notable for concerning antimicrobial resistance and virulence profiles. Rabbit represented the niche carrying the highest number of *mcr-1* genes (3 strains from one animal and two meat samples) among our collection. A recent Italian investigation [94] reported polymyxin (colistin) as a widely used antimicrobial in rabbit farms even though two reports indicated colistin was not a common treatment in rabbit breeding systems [41,95]. Resistance to colistin has been previously reported in breeding rabbits in Europe, supporting the data from the third report [96]. To our knowledge, this is the first report of *mcr-1* identified in rabbit meat products. This finding suggests rabbit farms and meat should be investigated as a potential reservoir of *mcr-1* and as a vector for its transmission. Concerningly, eight rabbit strains with ST20 (4/23) and ST40 (4/23) also harbored extensive virulence profiles. These profiles displayed co-presence of both ExPEC and IPEC VAGs and did not correspond to the usual pathotype observed in these lineages. ST20 and ST40 are reported as human diarrheagenic pathogens [97], often producing Shiga toxins [98,99,100]. The emergence of new hybrid pathotypes could represent a serious threat to public health [101,102], as witnessed in several previous outbreaks [103,104]. Further studies are needed to better understand this phenomenon and the potential animal sources involved in hybrid pathotype evolution and diffusion.

Poultry source strains (11/25; 44%) belonged to some of the most prominent ExPEC sequence types (ST131, 1; ST69, 1; ST10, 1; ST23, 3; ST117; 5), all of which were isolated from poultry meat and are previously reported in this niche [25,105,106,107]. ExPEC lineages carried the highest number of VAGs (mean = 22) among poultry source and a high number of ARGs (mean = 5). ST117, a known avian pathogenic extraintestinal *E. coli* (APEC) lineage and a human pathogen [25,106,108] was the most common sequence type among poultry source. Poultry meat is a frequently investigated source of ExPEC *E. coli*, which can be transmitted to consumers through food consumption [22]. Fecal contamination of poultry carcasses during slaughter could allow potential ExPEC diffusion through the food chain [109,110]. Our findings underline the role of poultry as a source of potential ExPEC lineages and the importance of related meat as an ExPEC carrier to humans.

Although wildlife is not as widely scrutinized as food animals for potential threats to human health, a relatively high number of VAGs (mostly associated to ExPEC pathotype) were observed in our wild animal (mean = 11) and wild boar (mean = 8) isolates, with 10/47 (21.3%) isolates carrying ≥15. Notably, four strains belonged to some of the predominant ExPEC lineages (2, ST23; 1, ST117; 1, ST648). The ST648 strain carried the highest number of VAGs (37) in the entire collection. ST648 has been isolated from wild birds [111], humans, surface water, fish, vegetables and companion animals [112,113], has been linked to disease in both human and animals (pets, horses and wildlife) (https://enterobase.warwick.ac.uk/, accessed 19/10/2019) and is frequently reported as an ESBL gene carrier [113,114].

### 3.5. Conclusions

Briefly, our study reaffirmed the role of food-producing animals as a reservoir of potential zoonotic pathogens, with variable antimicrobial and virulence traits among the sources investigated. In particular, rabbits and poultry represented the most concerning sources, carrying the highest number of ARGs and VAGs. Poultry was associated with potential ExPEC strains. Meanwhile, rabbits were a source of potential hybrid *E. coli* pathogens and carriers of *E. coli* with *mcr-1*.

It should be noted that our study has two important limitations. Firstly, the small sample size of each source (in particular of companion animals) prevented accurate quantitative comparison between antimicrobial and virulence profiles and the evaluation of possible ARGs and VAGs transmission routes between environments. Secondly, our data originated from short read sequencing analysis. Therefore, we could not determine the location of all ARGs identified and their association with MGEs. Moreover, we were not able to establish similarities/dissimilarities between F plasmids, potentially responsible for VAGs and ARGs carriage in our collection.

Despite these limitations, our study provided basic information about AMR and virulence determinants circulating in various environments in Italy. Further investigations can add a deeper understanding of AMR and virulence epidemiological traits in Gram-negative bacterial populations of different settings.

## 4. Materials and Methods 

### 4.1. Sampling

In the period between November 2010 and May 2018 a total of 300 commensal *E. coli* were collected from 12 different food, animal and human sources (dairy, beef, wild boar, rabbit, poultry, swine, vegetable, fishery, mollusc, wild animal and human), mainly in the Emilia Romagna region of Italy.

Food samples (chicken, rabbit and swine meat products, vegetables and fish) were collected from major supermarkets located in the province of Bologna and from the educational abattoir of the Veterinary Sciences Department (University of Bologna), during slaughtering procedures (sponge of beef carcasses). Milk, cheese and milking system filter samples, collected in a previous study by our research group, were included in the project.

Among animal samples, feces (cat and dog) and cloacal swabs (poultry) were collected from healthy individuals, that had not received antimicrobials in the month prior to the collection. Wild boar diaphragm samples were supplied by Istituto Zooprofilattico Sperimentale della Lombardia e dell’Emilia Romagna (IZSLER), Bologna chapter.

Collection of human samples were approved by the University of Bologna Bioethics Committee under internal protocol number 0252770. Human feces were collected from healthy volunteers that were approached for recruitment in person. These participants had not had any antimicrobial treatments in the month prior the collection. 

All food, animal and human samples were carried to the laboratory in aseptic conditions and analyzed within two hours from the time of gathering.

IZSLER Bologna, Forlì and Reggio Emilia chapters contributed to the strain collection with 97 hypothetical *E. coli*, isolated from different animal sources (mollusc, wild animal, rabbit, beef, companion animal, swine). Among these isolates, 12 strains of companion animal origin were isolated from diseased animals and were therefore excluded from the study to avoid the possibility of including pathogens.

The final collection was therefore characterized by 25 *E. coli* for each source (except for companion animal, n = 13), for a total of 288 strains.

A comprehensive description of the strain collection is provided in Supplementary Material, Table S1.

### 4.2. Bacterial Isolation

Twenty g of food sample (in the case of carcass sponge, each one separately) and wild boar diaphragm were placed into sterile blender bags, diluted in 180 mL of sterile EC-Broth (Oxoid, Basington, UK) and macerated in a stomacher for 1 min. Samples were incubated overnight at 37 ± 1 °C. Fecal samples (1 g each) were diluted (1:10) in peptone water (Oxoid, Basington, UK) and homogenated by vortexing.

Ten µL of overnight culture in EC-Broth (Oxoid, Basington, UK) and 10 µL of feces solution (or directly in the case of the cloacal swabs) were streaked onto MacConkey (Oxoid, Basington, UK) and Levine (Oxoid, Basington, UK) agar plates and incubated for 18–24 h at 37 ± 1 °C. For all the samples, lactose fermenting colonies were collected and assessed for Gram stain and standard biochemical test (indole probe). *E. coli* ATCC 25,922 was used as a control strain.

### 4.3. Storage

All strains were freshly cultured on TSA (Oxoid, Basington, UK) for 18–24 h at 37 ± 1 °C and stored at −80 °C in cryoprotective medium, composed of Tryptone Soya Broth (Oxoid, Basington, UK) and 20% glycerol.

### 4.4. DNA Extraction and Isolate Identification

Bacteria were grown from glycerol stocks on Tryptone Soya Agar (TSA) (Oxoid, Basington, UK) plate overnight at 37 ± 1 °C. Genomic DNA was extracted using a commercial kit (DNeasy Blood and Tissue Kit, Qiagen, Hilden, Germany), following the manufacturer’s instruction.

A multiplex PCR targeting four genes (*lacY, lacZ, uidA, cyd*) was used for *E. coli* identification, following the method described by Horakova et al. (2008) [115].

The PCR amplification was performed in a reaction volume of 10 µL containing 5 µL REDExtract-N-Amp PCR ReadyMix (Sigma-Aldrich, St Louis, MO, USA), 0.25 µL primers (10 pmol) and 1.5 µL DNA. 

The following amplification parameters were applied: initial denaturation at 94 °C for 3 min, 30 cycles of denaturation at 94 °C for 30 s, annealing at 58 °C for 25 s, elongation at 72 °C for 30 s and a final extension at 72 °C for 3 min.

The amplified products were loaded onto a 2% agarose gel containing Syber Safe DNA Gel Stain (Invitrogen, Carlsbad, CA, USA) and run in 1X TBE buffer at 100 V for 1 h.

PCR fragments were visualized with a UV transilluminator. A pUC19 DNA/MspI (Hpall) Marker (Thermo Fisher Scientific, Waltham, MA, USA) was loaded on each gel as a DNA size standard. *E. coli* ATCC 25,922 DNA was present in every run as a positive control strain. Strains showing PCR products of 463 bp, 393 bp, 319 bp and 264 bp were considered *E. coli*.

### 4.5. WGS and Assembly

Library preparation was performed using the Nextera Flex library preparation kit (Illumina, San Diego, CA, USA). Briefly, genomic DNA was quantitatively assessed using an Invitrogen Quant-iT picogreen dsDNA assay kit (Thermo Fisher Scientific, Waltham, MA, USA). The sample was then normalized to a concentration of 1 ng/µL and 10 ng of DNA was used for library preparation. After the tagmentation step, DNA was amplified with 12 PCR cycles using the facility’s custom designed i7 and i5 barcodes as previously described [59].

Due to the number of samples, the quality control for the samples was done by sequencing a pool of samples using MiSeq V2 nano kit—300 cycles (Illumina, San Diego, CA, USA). Briefly, 3 µL of each library was pooled into a library pool, cleaned up using SPRI beads following the Nextera Flex clean up and size selection protocol. The pool was then sequenced using MiSeq V2 nano kit (Illumina, San Diego, CA, USA). Based on the sequencing data generated, the read count for each sample was used to pool libraries at a different amount to ensure equal representation in the final pool and to discard failed libraries (i.e., libraries with less than 100 reads). The final pool was then sequenced on Illumina NextSeq 500, 2 × 150 bp at Ramaciotti Centre for Genomics (University of New South Wales, Australia).

Sequence read quality was assessed using FastQC version 0.11.5 (http://www.bioinformatics.abraham.ac.uk/projects/fastqc/). Illumina raw reads passing quality control were assembled into draft genome sequences using Shovill v1.0.4 with default settings and trimming options (https://github.com/tseemann/shovill).

Sequencing reads were deposited in the National Center for Biotechnology Information (NCBI) database with study accession number PRJNA528851. Accession numbers for each sample are listed in Supplementary Material, Table S1.

### 4.6. Gene Identification, Serotyping, Phylogrouping and Multilocus Sequence Typing

All gene screening was performed using ARIBA [116] as well as reference sequences from a variety of publicly available databases. Resistance, virulence, plasmid-associated genes and OH antigen genes were obtained from ResFinder [117], VirulenceFinder [118], PlasmidFinder [119], and SerotypeFinder [120], respectively.

Other sequences of interest (insertion sequence elements, AMR and virulence associated gene sequences), available at https://github.com/maxlcummins/E_coli_customDB and not present within the previous databases were also screened. Moreover, different efflux pump/biocide resistance gene sequences collected from GenBank were considered (Supplementary Material, Table S2). Pointfinder [121] was used to establish chromosomal mutation in *gyrA/B*–*parA/C/E*, *ampC* and *pmrA/B*, predicting phenotypic resistance to quinolones, AmpC-type cephalosporins and colistin, respectively.

*E. coli* phylogroups were determined using the Clermont scheme [122], meanwhile the Achtman scheme was used to evaluate *E. coli* multilocus sequence types (MLST) (http://mlst.warwick.ac.uk/mlst/). ARIBA results were then processed and summarized with ARIBAlord (https://github.com/maxlcummins/ARIBAlord).

### 4.7. Phylogenetic Analysis

Maximum-likelihood phylogenetic distances between genomes were analyzed using the PhyloSift pipeline [123], and a tree was generated using FastTree2 [124]. The tree was constructed using FigTree v1.4.4 (http://tree.bio.ed.ac.uk/software/figtree/) and iTOL (https://itol.embl.de/).

## Supplementary Materials

The following are available online at https://www.mdpi.com/2079-6382/9/11/782/s1, Table S1: Metadata, virulence and AMR profile, MGE carriage of the collection Table S2: Efflux pump/biocide database used in the present study, Table S3: Chromosomal mutations identified in the collection, according to Pointfinder analysis, Table S4: Efflux pump/biocide resistant gene carriage.
